# Integration of Mental Health Support Teams in COVID-19 Units within French General Hospitals: A Qualitative Study

**DOI:** 10.5334/ijic.6454

**Published:** 2022-11-28

**Authors:** Nicolas Coustals, Ana Moscoso, Noël Pommepuy, Jordan Sibeoni

**Affiliations:** 1 Department of Child and Adolescent Psychiatry, Ville-Evrard Hospital, Neuilly-sur-Marne, France; 2 Department of Child and Adolescent Psychiatry, Robert Debré University Hospital, Paris, France; 3 Service Universitaire de psychiatrie de l’adolescent SUPADO, Argenteuil Hospital Centre, Argenteuil, France; 4 ECSTRRA Team, UMR-1153, Inserm, Paris Cité University, Paris, France

**Keywords:** integrated care, mental health, COVID-19, epidemic, person-centered care, family-centered care, soin intégré, santé mentale, COVID-19, épidémie, soin centré sur la personne, soin centré sur la famille

## Abstract

**Introduction:**

This study aimed to explore the lived experience of mental health professionals (mhPs) who had been redeployed on support teams (MHSTs) implemented in general hospital for patients with coronavirus disease 2019 (COVID-19) and their families, in order to scale up mental and physical health care integration in times of epidemic crisis.

**Methods:**

This multicentered qualitative study followed an IPSE (Inductive Process to analyze the Structure of lived Experience) research design. MhPs’ recruitment took place in three general hospitals of Seine-Seine-Denis department, in Paris suburbs (France).

**Results:**

Twenty-two participants were included. Data analysis produced three central axes: 1) the mhP in the epidemic crisis, underlying how participants confronted the unknown and adapted; 2) retrieving fundamentals of support therapy, that were: being present and listening, bonding with patients’ families, and ensuring care continuity; and 3) moving forward with other health professionals, highlighting the collaborative work they developed and experienced.

**Discussion:**

The epidemic prompted mhPs to rethink the values likely to guide the integration of their intervention with other individual and organizational care stakeholders, at different levels of health system. Normative integration based on shared appraisal of patients’ and families’ needs is highly required to overcome the multiple and sometimes contradictory health issues inherent in the crisis.

**Conclusion:**

Person- and family-centered approach of integrated care (IC) is essential to address fragmentation between mental and physical health care in times of epidemic crisis. Hospital and political leaders should support and draw from bottom-up mental health IC initiatives such as MHSTs, that embody this vision, in order to improve health systems preparedness for future crises.

## Abstract (fr)

**Introduction:**

Cette étude a visé à explorer l’expérience de professionnels de santé mentale (mhPs) redéployés sur des équipes de soutien (MHSTs) implantées à l’hôpital général pour les patients atteints par la maladie à coronavirus 2019 (COVID-19) et leurs familles, afin d’améliorer l’intégration des soins de santé mentale et physique en période de crise épidémique.

**Méthodes:**

Cette étude qualitative multicentrique a suivi le protocole de recherche IPSE (Inductive Process to analyze the Structure of lived Experience). Le recrutement des mhPs a eu lieu dans trois hôpitaux généraux du département de Seine-Saint-Denis, en banlieue de Paris (France).

**Résultats:**

Vingt-deux participants ont été inclus. L’analyse des données a fait émerger trois axes centraux : 1) le mhP dans la crise épidémique, soulignant comment les participants se sont confrontés à l’inconnu et se sont adaptés ; 2) retrouver les fondamentaux de la thérapie de soutien, qui étaient : être présent et écouter, faire du lien avec les familles des patients, et assurer la continuité des soins ; 3) avancer avec les autres professionnels de santé, sur la base du travail collaboratif que les participants ont développé et dont ils ont fait l’expérience.

**Discussion:**

L’épidémie a conduit les mhPs à repenser les valeurs susceptibles de guider l’intégration de leur intervention avec les autres acteurs du soin, individuels et organisationnels, à différents niveaux du système de santé. Une intégration normative basée sur une compréhension partagée des besoins des patients et de leurs familles apparaît primordiale pour répondre aux enjeux de santé multiples, et parfois contradictoires, inhérents à une telle crise.

**Conclusion:**

Une approche du soin intégré centrée à la fois sur le patient et la famille est essentielle pour palier la fragmentation des soins de santé mentale et physique en période de crise épidémique. Les décideurs hospitaliers et politiques devraient soutenir et s’appuyer sur des initiatives de soin intégré émanant du terrain telles que les MHSTs, qui incarnent cette vision, afin d’améliorer la préparation des systèmes de santé à de futures crises.

## Introduction

Over the past two decades, integrated care (IC) has been promoted as a means to improve access, quality and continuity of care, especially for people with complex medical needs and within contexts in which disconnection between different components of health systems occurs [1,2,3]. Albeit the diverse ways to define IC, the concept essentially refers to bringing together otherwise fragmented aspects of care [1], in order to meet people’s needs on a continuum of health promotion, disease prevention, diagnosis, treatment, disease management, rehabilitation and palliative care services [2]. Care integration can therefore be seen as a complex process that unfolds at multiple levels of health systems, from sociopolitical context (macro-level) to local care services (meso-level) and care stakeholders (micro-level), including health professionals as well as patients and their families [1,4].

Due to unprecedented pressures on health systems and their unpreparedness for such a pandemic, crisis of coronavirus disease 2019 (COVID-19) challenged care integration at all these different levels (i.e. macro, meso, and micro) [5,6,7,8], with particular risk of fragmentation between general and mental health care services [9,10,11]. In France, the first wave resulted in hospitalization of more than 90,000 people between March and June 2020 – 19% of whom went through intensive care [12] – and in death of more than 30,000 people [13]. Within a few weeks, the National Health System had to reorganize itself to cope with increasing flow of patients while dealing with limited staff resources, including mental health professionals (mhPs), and adopting drastic measures against viral transmission [14]. This sudden health system disruption led to prioritizing the answer to direct physical consequences of COVID-19 infection and prevention of epidemic spread, while access to mental health as other social and care services was reduced [9,11,15,16].

In the same time, feedback from first countries affected [17,18,19,20] as well as previous experiences of emerging respiratory infections, such as Severe Acute Respiratory Syndrome (SARS) and Middle East Respiratory Syndrome (MERS) [21,22,23], pointed out high risk of mental burden for patients affected by COVID-19 and their relatives. Anxiety and depression prevalence rates among patients with COVID-19 were estimated to be around 30% for the former and 40% for the latter [18], and around 20% for post-traumatic stress disorder [17]. Simultaneously, high proportion of patients’ relatives also suffered from psychological distress [24,25], especially when their loved ones were hospitalized in intensive care unit and/or when their visiting rights were restricted [25,26].

In mainland France, department of Seine-Saint-Denis (Paris suburb) was the most affected by the first epidemic wave, with an excess mortality of 134% between March and April 2020, versus 26% throughout the country [27,28]. Local psychiatric hospital, with the help of established Consultation-liaison psychiatry services, decided to react by creating new mental health support teams (MHSTs) integrated in three general hospitals of the department, dedicated to patients admitted for COVID-19 infection and their families.

Many crisis support devices have been deployed worldwide to meet the psychological needs of people facing the disease [16,29,30,31,32]. Yet, no qualitative study has ever investigated the experience of mhPs involved in such integrated services in general hospital, neither during the COVID-19 pandemic nor during other emerging respiratory infection. Our study aimed to fill this gap and to explore the lived experience of these professionals.

## Methods

This multicentered qualitative study followed the IPSE (Inductive Process to analyze the Structure of lived Experience) approach, a practical method of qualitative research informed by constructivism, phenomenology, and grounded theory [26]. IPSE is based on an inductive process of exploration and analysis of participants’ lived experience. Five stages, as stated below, organize the entire research process.

The research complies with French regulations governing observational research involving professionals (declaration of compliance with the CNIL reference methodology MR004 and entry in the register of such research hosted by Health Data Hub website). All participants provided informed consent before inclusion. The report of this study adheres to the COREQ guidelines (see supplementary material) [33].

### 1. Setting up a research group

Our research group included one male resident in Psychiatry (N.C.), and three child and adolescent psychiatrists, one female (A.M.) and two males, one of whom created the MHSTs (N.P.) while others (A.M. and J.S.) had no prior knowledge of them. N.P. is the head of an hospital psychiatric department for children and adolescents, and trainer in family therapy. J.S. has a PhD in public health specialized in qualitative health research. A.M. is working in an university hospital in Paris. N.C. was working in the psychiatric department of N.P. at the time of the study, with personal interest in family therapy and consultation-liaison psychiatry. Researchers were diverse in their age, sex, and professional background. They worked continuously on researcher’s reflexivity during open and regular discussions.

### 2. Ensuring study originality

A member of the research team, expert in qualitative research (J.S.), performed a rapid review in three data bases (PUBMED, PsychInfo and SSCI) according to a search algorithm specific to each base, all through June 2020. Preliminary research identified several articles from which we selected key words, a mix of free-text terms and thesaurus terms that refer to integrated care, mental health, pandemic and epidemic respiratory infection, to collect studies indexed in the databases and ensure study relevance and originality. After collecting the references and eliminating duplicates, the same author subsequently read the titles and abstracts to assess their relevance to our topic and read in full potentially relevant articles. No qualitative study exploring the experience of mhPs who would have specifically supported patients hospitalized for COVID-19 or their families was found.

### 3. Recruitment and sampling, aiming for exemplarity

Sampling strategy was purposive, with maximum variation [34], in order to select professionals that differed in position, sex, age, family status, years of experience, and usual practice. This strategy aims for exemplarity, that is, including participants who have experienced archetypal examples of the studied situation and might progressively enrich findings of ongoing analysis with new narratives. We first reached the MHSTs’ coordinators of the three hospitals to identify all professionals involved, then contacted them and conducted a preliminary interview to present the study and verify that they met the predefined inclusion criteria. Inclusion criteria were: 1) having worked as a psychologist, psychiatrist, or health manager in one of the three hospital MHSTs of Seine-Saint-Denis department; and 2) speaking French fluently. The only exclusion criterion was being on sick leave.

Sample size was not defined in advance but determined by data saturation according to the principle of “theoretical sufficiency” [35]: inclusion of new participants continued until analysis of new material no longer yielded new findings, that is, data collection and analysis were complete when researchers considered that the obtained axes of experience provided a sufficient explanatory framework for collected data. Saturation is a key criterion for validity in qualitative research, as it ensures in-depth study of the concerned phenomenon and suggests that further interviews are unlikely to produce new findings.

### 4. Data collection, access to experience

From June through September 2020, one researcher (N.C.) conducted single interviews with all included participants. The researcher had no relationship with participants prior to study. The researcher met participants successively and collected social/demographic data – sex, age, profession, professional experience (years), and usual activity service(s) – to facilitate subsequent research. Information about the research was given (objectives, methodology, future publication). All participants agreed to participate in the study and gave written consent.

A few days after the first meeting, the researcher conducted a single semi-structured interview with each of the participants, in their workplace, in a one-to-one setting, using an *open-ended approach* structured by areas to explore. These areas (Table 1) were determined by the research team from analysis of two pilot interviews. The researcher used an interactive conversational style. Interviews lasted 45 to 75 minutes. They were audio recorded and transcribed into anonymised verbatim, including participants’ expressive nuances. These transcripts were then analysed, without being returned to participants for comments or corrections. The interviewer took field notes after every interview to better work on reflexivity during the research group meetings.

**Table 1:** Exploration areas.


1) Motivations for participating in MHST, expectations*

2) Supportive work with patients, their families and other health professionals

3) The disease, risk of viral transmission, end-of-life situations

4) Work with other members of the MHST

5) Work with other health professionals in somatic departments

6) Difficult things, supports, what to retain from this experience*


MHST: Mental Health Support Team. * Area added after pilot interviews.

### 5. Data analysis

Analytic procedure followed the IPSE approach [36], unfolding at both individual and collective levels. In the individual procedure, two qualitative researchers (N.C. and J.S.) independently and simultaneously conducted systematic descriptive analysis aimed at conveying each participant’s experience. For each interview, this involved: 1) listening to the recording twice and reading it three times; 2) exploring the narrated experience word by word, that is, cutting up the entire text into descriptive units; 3) regrouping these descriptive units into categories. These stages were carried out with the help of QSR NVivo 12 software.

All researchers familiarized themselves with data through listening to and reading all interviews as many times as necessary. After analysis of five interviews, they met four times for two hours in order to conduct: 1) the structuring phase, that is, to regroup categories into axes of experience; these axes being constructed such that each can be linked to its subjacent categories, and then to determine structure of lived experience characterized by central axes; 2) the practical phase, a process of triangulation with data in literature, so to identify original aspects of the results.

We used several criteria to ensure analytic rigor and reliability of the results: triangulation, attention to negative cases, and reflexivity throughout the research group process. Participants provided feedback on the findings.

## Results

Twenty-two participants were included in this study to reach data saturation (Table 2), out of twenty-seven health professionals involved in the MHSTs. The five professionals not included in the study were approached but did not yet respond to researchers’ requests when data analysis reached theoretical sufficiency.

**Table 2:** Sociodemographic characteristics of participants and length of interviews.


PARTICIPANT	SEX	AGE (YEARS)	PROFESSION	PROFESSIONAL EXPERIENCE (YEARS)	USUAL DEPARTMENT(S) OF ACTIVITY (TYPE OF CARED POPULATION)	LENGTH OF INTERVIEW (MINUTES)

P1	F	32	Psychologist	6	CLP (children and adolescents) MPC (children)	72

P2	F	42	Psychologist	15	CLP (children and adolescents) MPC (adolescents)	55

P3	F	42	Psychologist	17	CSPCA (adults)	55

P4	F	34	Psychologist	11	CLP (adults)	50

P5	F	33	Psychologist	9	CSPCA (adults)	40

P6	F	32	Psychologist	9	CLP (children and adolescents)	42

P7	F	29	Psychiatrist	4	DH (children with ASD) MPC (children)	45

P8	F	34	Psychiatry resident	3	CLP (children and adolescents) MPC (children)	54

P9	M	28	Psychiatry resident	3	DH (children with ASD) MPC (adolescents)	51

P10	M	48	Psychiatrist	19	MPC (children and adolescents)	57

P11	F	40	Psychiatrist	11	DH (children with ASD) MPC (children and adolescents)	63

P12	M	40	Psychologist	9	MPC (children)	63

P13	F	39	Psychologist	15	MPC (adults)	61

P14	F	37	Psychologist	13	CLP (children and adolescents) DH (children with ASD) MPC (children and adolescents)	66

P15	F	39	Health manager	12	CLP (children and adolescents) DH (children with ASD) MPC (children and adolescents)	74

P16	M	42	Psychiatrist	12	CLP (adults)	60

P17	F	43	Psychologist	15	MPC (children and adolescents)	62

P18	F	51	Psychologist	4	MPC (children and adolescents)	52

P19	F	35	Psychiatrist	9	CLP (children and adolescents) MPC (children)	70

P20	F	50	Psychologist	15	CLP (adults)	62

P21	F	38	Psychologist	3	CLA (adults)	71

P22	F	62	Psychologist	15	MPC (adults)	57


ASD: Autism Spectrum Disorder; CLA: Consultation-Liaison in Addictology; CLP: Consultation-Liaison in Psychiatry; CSPCA: Care, Support, and Prevention Center in Addictology; DH: Day Hospital; F: female; M: male; MPC: Medico-Psychological Center.

Data analysis produced a structure of lived experience based on three central axes: 1) The mhP in the epidemic crisis; 2) Retrieving fundamentals of support therapy; and 3) Moving forward with other health professionals.

Relevant quotations (from interview transcripts, translated from French into English for sole purpose of this article) are presented in Table 3.

**Table 3:** Illustrative quotes (right column) by axes and subthemes of experience (left column).


THE MHP IN THE EPIDEMIC CRISIS	

Meeting the needs	Q1: “I had the motivation to make myself useful because I could see that it was panic on board, that it was complicated in hospitals, the hardest thing for me would have been to do nothing during this period… It was also our place as caregivers to be where it was needed”

	Q2: “At the very beginning we were waiting for demand, which did not arrive, so we broadened our care offer a little, we regularly went to handovers [with clinical staffs], we systematically called families of patients who arrived in the unit, and we went to see patients who needed it […] We saw how it was [in COVID-19 units], it was like war over there, so we can understand that they didn’t have the reflex to call us”

	Q3: speaking of having gone to see hospital’s mortuary service: “We wanted to get concrete information on how things were really going in the context of COVID, because we said to ourselves that families were quite lost, that they were certainly going to ask us questions, and we wanted to be able to answer them and help them with details of death aftermath [of their loved one]”

Confronting oneself to the unknown	Q4: “I had a lot of questions about hospital environment, work of psychologist in hospital, because I had done very little of this before, […] I had a lot of positioning questions about that”

	Q5: “I wasn’t really afraid of the virus, but more of dealing with a fragile, weakened person, fear of not having the right words, of disturbing, of not succeeding in relieving suffering”

	Q6: “The first image of my confrontation with this service, that is we arrive and we have a coffin that comes out in the other direction, we were perhaps on the 3rd or 4th day of work, and there I said to myself okay, that’s what we’re going to do right now, I don’t know if I had anticipated that”

Adapting and being efficient	Q7: about telephone support for the daughter of a deceased patient: “I offered to see her, but it was too complicated, […] we didn’t know how to do it, she was all alone, whereas usually [outside COVID-19 period] we are surrounded [during grief]”

	Q8: “At the beginning we received a lot of anger and guilt from teams, this wasn’t our main mission but well, we were there so we did it”

	Q9: “We tried things, we saw that some worked, others did not, it was a real brainstorming the first week, […] we were launched but the design [of our intervention] really came gradually, not at all upstream”

**RETRIEVING FUNDAMENTALS OF SUPPORT THERAPY**	

Being present and listening	Q10: “Finally I used my clinical sense, what I knew how to do, just listening, being in a presence for patients, […] doing simple things, not complicating oneself”

	Q11: “Listening it’s already huge, just validating, saying yeah you feel that that’s okay, this is already quite a lot”

	Q12: “We tried to put in place as many things as possible, even a little banal, […] knowing if they were able to call their family, if they did eat, if they were thirsty”

	Q13: “Sometimes I took their hands, I found them really alone, isolated, the least we could do was to approach them anyway, not to stay more than a meter as it was planned”

Bonding with families	Q14: “We made a lot of links between patients and their families, sometimes we held the phone, informing the family that we had seen the patient, we made this link a lot between the patient and his family”

	Q15: “We reassured patients who sometimes didn’t understand why their children didn’t come, no this is not that they’ve forgotten you, this is that visits are not possible”

	Q16: about calls to the family of a patient: “I transmitted what I observed from Mrs. B., who certainly was unconscious but who seemed rather calm, appeased… I tried to convey something of how she was in the room”

Ensuring care continuity	Q17: about clinical staffs of COVID-19 units: “We were present with them at the morning meeting where we took information on patients, then we saw each other again during the day to give a brief, […] the state of health of patients moved so fast that I needed to see the doctor again to know where the situation was”

	Q18: about a patient with COVID-19, leaving maternity ward: “We had zero relay once she was released from hospital, so I called her 2–3 times a week to accompany her return to home, time for anxiety to subside, for her to put herself in her maternal role”

	Q19: about another support device for bereaved families: “We said to ourselves, that’s a shame that people don’t benefit from this system, which is very complementary to ours, […] so we called them back to get some news and to tell them that this device existed”

**MOVING FORWARD WITH OTHER HEALTH PROFESSIONALS**	

Relying on teamwork	Q20: “It was both the support of the team in its experiences and in its practice, and at the same time at a more personal level, at the level of this benevolence that we had for each other, I find that it was a mixture of the two”

	Q21: “It was not a completely assembled device that we had to fit into, but it was something that we had to build together, and that’s what we did, and that was rather interesting, […] that everyone puts a little of his own”

	Q22: “I had a lot of admiration for colleagues who usually worked in [psychiatric] liaison, whom I found quite comfortable to initiate discussion both with other caregivers and patients… They served a bit as a model, a guide at the start”

Learning to work along with teams of physical health care	Q23: “There were patients who did not feel well and who could not verbalize it, anxious patients or rather sad patients, the team was kind enough because they really anticipated: when they found that a patient was a little more withdrawn or a little sadder, they called us”

	Q24: “From the moment we went to the staff at 9am, we were part of the team, from then on it was much easier to interact with doctors”

	Q25: about exchanges with other caregivers: “We would arrive by saying do you want us to discuss a little, and in the end it lasted a little longer than classic pose, and that’s what often works in the end, informal times, it’s easier than when it’s organized in a meeting”

Supporting the relational and human aspect of care	Q26: “The idea was that there is a place in medicine for psyche, and that it goes well, that we get there well, and my feeling with this experience of the MHST, that’s it was well done, it was well done”

	Q27: “Being there, I think it created a link, humanization, because caregivers were in death, in care, in terrible things”

	Q28: “I think it was also somewhat our role to support these team reflections on how we take charge, how we mentally support families and patients”


COVID-19: Coronavirus disease 2019; mhP: mental health professional; MHST: Mental Health Support Team.

### 1. The mhP in the epidemic crisis

#### a. Meeting the needs

Participants described the need they felt to make themselves useful and respond quickly to the critical psychological needs they perceived in people facing COVID-19 (Q1). Many considered that being proactive was essential to receive requests for support and intervene promptly with patients, families or even caregivers who needed it (Q2). They emphasized how important it was to be easily available to other caregivers through regular presence in the wards and use of single telephone number. Several participants pointed out that they also anticipated very concrete requests such as organization of funeral rites for deceased patients (Q3).

#### b. Confronting oneself to the unknown

Participants very often used the expression “the unknown” to describe the beginnings of their activity, especially regarding the precise missions of the MHSTs, working in unfamiliar hospitals or wards (Q4), with physically ill people (Q5), and also being confronted with death (Q6), end of life, bereavement, psychological trauma, and risk of being infected and contaminating their loved ones. Some also questioned their level of knowledge or competence to intervene in these situations.

#### c. Adapting and being efficient

Participants emphasized the importance of adaptability throughout their activity in the team. They stressed the need to be responsive, rapid, and efficient in setting up these organizations as well as in their daily interventions (Q7). Very often, they felt that their support to somatic wards’ professionals had been essential even if it was not part of their initial mission (Q8). They also stressed that they had been very careful not to disrupt their functioning, both at the individual and team levels. Finally, they experienced that the scope and means of their interventions had become clearer as their activity progressed (Q9).

### 2. Retrieving fundamentals of support therapy

#### a. Being present and listening

Participants very often used both words “presence” and “listening” to describe their support activity. They underlined the simplicity of these attitudes (Q10), the importance of accepting all the expressed experiences, including thoughts and feelings of anger, hostility, and hopelessness, showing understanding (Q11) and being able to “normalize” them, as in the case of frustration due to family presence restriction or visual hallucinations sometimes occurring after coma. They reported that their exchanges with patients took place in a certain relational warmth, with concrete and practical attentions towards them (Q12), physical proximity and sometimes tactile gestures which, in their view, prevailed over distancing rules (Q13), though always keeping their masks and other classical COVID-19 clothing for caregivers at hospital.

#### b. Bonding with families

Participants emphasized their willingness to address the anguish of separation between patients and their families that arised from restriction of visiting rights in COVID units, by helping them to maintain telephone/video contact (Q14), and reassuring each of them about the psychological state of the other(s) (Q15, Q16). Many felt the need to welcome or accompany families within the hospital when they could come, and found it difficult that the latter could not be more present with patients, especially in end-of-life situations.

#### c. Ensuring care continuity

Participants stressed the importance of information handover both within MHSTs and with teams of COVID-19 units (Q17), including single telephone line to centralize requests, and referring mhP(s) for each monitored patient/family. They often stated that it was useful to extend patients’ follow-up after their discharge (Q18) and that of families if their loved one died (Q19). Very often, they considered themselves and their interventions as an essential *stepping stone* to refer and guide patients, families, and health professionals to specialized psychiatric services or other crisis support systems if necessary (Q19).

### 3. Moving forward with other health professionals

#### a. Relying on teamwork

Participants highlighted how essential it was to intervene as a team (Q20). In their narratives, they used “We” as much as “I”, valued team cohesion and the fact that many facets of their work were co-constructed within the group (Q21). They also valued the support provided by experienced medical coordinators and/or CLP professionals (Q22). They underlined the importance of benevolence, mutual aid, and trust within the team (Q20), along with many moments of shared pleasure, relaxation, and humor.

#### b. Learning to work along with teams of physical health care

Participants considered that health professionals directly working within COVID-19 units were at the best place to perceive patients’ psychological needs (Q23). It was therefore essential to work closely with them. They often highlighted that they needed to approach them at the right time, given their pace of activity and availability, and to explain the support system to them carefully. They tried to multiply opportunities to exchange views with them during both formal times (e.g., medical staffs) (Q24), and more informal ones, such as their so-called “rounds” in different COVID-19 units (Q25).

#### c. Supporting the relational and human aspect of care

According to participants, it was crucial to take all psychological consequences of the disease into account during patients’ treatment, despite the concurrent emergency for their physical health (Q26). Professionals of somatic care, participants stated, were in fact reassured by their presence and actions with patients and families, which could secure holistic and humanistic approach of care in challenging times of pandemic (Q27). Some participants reported that their interventions also had an impact on those professionals and enabled them to display and use their own ability to provide psychological support to patients and families (Q28). In the latter case, they underscored that their function with other caregivers could be limited to supervision or aid to thinking, even in such difficult situations as bereavement.

## Discussion

The pandemic outbreak brought unprecedented pressures on French health system as on others worldwide [13,37,38,39]. Yet, our findings describe pre-existing weaknesses in care integration culture and fostering, especially the disconnection between national regulations (e.g. lockdown, social distancing, shut-down of health care services considered as non-priority), hospital policies (i.e. withdraw of psychologists from the wards, restriction of family visiting rights), and local initiatives of health care services leaders, without any top-down directive in favor of integrated care maintenance in the field of mental health. This observation is in line with Bajeux et al. findings in their review on the evolution of care integration for older people in France between 2010 and 2020: these authors state that despite improvements in the culture of professional collaboration and creation of regional health agencies ten years ago, many programs of IC remain experimental with established pathways concerning only very few conditions (renal insufficiency before dialysis and diabetes) [40]. In the field of mental health, several voices also stressed the non-adaptation to psychiatry of emergency plans for health care facilities – called White Plans – established by French law in 2004 and activated during the first wave of COVID-19 epidemic, calling for inclusion of psychiatrists in the development of these plans and for creation of psychological and psychiatric Whites Plans [16].

Apart from functionals aspects required by health services integration (e.g. inter-organizational agreements, funding, human resources, technical and informational support) [3], in our results, participants insist on the importance of a shared vision of people’s health needs and care objectives, i.e. the importance of *normative integration* between the different levels and actors of health systems [3,41,42]. Common values are needed to guide care integration, even more in times of epidemic crisis due to multiple and sometimes contradictory health issues, as well as general reorganization of care networks [5,8,40]. In that regard, our findings show that MHSTs implementation success in general hospital depended mainly on a shared recognition of the psychosocial consequences, for hospitalized patients and their relatives, of both the disease and restrictive measures taken to fight against viral transmission. This time of epidemic crisis revealed the discrepancy in the conception of care according to health professionals – advocating for an holistic and person/family-centered approach of care – and governmental and hospital policies – that have the tendency, at least initially, to prioritize urgent physical care and reduction of contagion risk. Our results suggest that MHSTs implementation could also enable to better integrate patients’ and families’ needs and to facilitate communication between care providers and administrations from both general and psychiatric hospitals.

Adopting a person-centered approach appears essential to guide care integration, placing users’ care experience and response to their needs at the center of the care objectives described by the «Triple Aim» model, alongside improving population health and cost reduction [1]. It entails holistic vision of care, acknowledging the complex and evolving nature of people’s needs across dimensions of physical, psychological and social well-being [2,5,43]. Based on the Social Support Needs framework developed by James House [44], a recent study developed a five-dimension evolutive model of these needs (namely *informational, emotional, appraisal, instrumental* and *spiritual*), from admission into intensive care unit until readaptation into community [45], and concluded that requirement to meet such needs was the involvement of multiple care providers, including nurses, clinicians, but also relatives, with increasing need of support from the latter along the recovery process [45].

Collaboration with family is a central component of person-centered integrated health services [2], and our results strengthen previous findings showing that health systems were not prepared to maintain it during a crisis such as COVID-19 pandemic [26,46,47,48]. Adopting an integrated care approach more explicitly person- *and* family-centered could address this issue, as our results show and as it is already the case in certain medical specialties outside epidemic context (e.g. pediatrics [49,50], child psychiatry [51,52,53], critical care [54,55], and palliative care [56]).

In times of health crisis, however, this approach must consider the limited time and attention caregivers have to learn new skills, as well as restrictive measures on social contact [57]. During COVID-19 pandemic, many intensive care units and other COVID-19 departments therefore set up special teams – made of professionals without specific training in mental health (e.g. medical students, nurses, physicians) – to maintain communication between patients, their loved ones, and medical staffs, with enhanced use of telehealth [58,59,60,61,62,63,64]). While such teams could provide information to families, promote calls with patients and deliver basic emotional support, they could not ensure specialized mental health care or supervision of other health professionals. Our results suggest that implementation in hospital wards of support teams composed by mhPs is a better option to achieve full integration between physical and mental health care for patients and families.

Finally, our findings encourage promotion of mental health IC initiatives emerging from the field, when only top-down decision-making process appears too rigid and does not fit care goals and values of users and/or health professionals. During the pandemic, the ability for leadership to listen to solutions suggested by front-line workers has been highlighted as critical to ensure efficient, flexible, and timely health system response to IC needs [65]. In line with complex adaptative systems theory applied to health [66,67,68,69], our findings also suggest that, in times of health crisis, self-organizing ability of IC service is an important factor for the success of its implementation. This strengthens previous findings stating that leadership should mainly guarantee environment enabling self-organization, through balance between reliable IC structure and flexibility to adapt to local context [65,70]. At the political level, new reform launched in September 2020 in response to COVID-19 outbreak in France – called “Ségur de la Santé” (referring to location of Ministry of Health in Paris) – laid the stones for the success of such model, with both national financial effort and promotion of territorial projects in mental health, relying on enhanced collaboration between proximity actors [71]. Future hospital and governmental policies should draw from bottom-up IC experiences such as MHSTs of the present study, in order to support further extension of mental and physical health care integration.

### Strengths and limitations

This is the first study exploring the experience of mhPs involved in mental health support teams especially launched at general hospital during the COVID-19 pandemic. The method we applied is rigorous and tailored for medical research [36], and this report meets the COREQ guidelines’ criteria [33].

Nevertheless, it also presents several limitations that must be taken into account. First, it took place in France, and caution is required in transposing our results to other places given that mental health support in general hospital depends strongly on the organization of the healthcare system as a whole, along with other territorial socioeconomic conditions and policies. Second, it did not investigate the experience of the users of these new care devices: patients, families, and somatic wards’ caregivers. For instance, this limitation could explain that patients’ spiritual needs identified in other studies during COVID-19 and SARS pandemics [72,73,74,75,76], as outside following critical illness [77], did not appeared in our results. Further research involving these stakeholders would be useful and relevant to enrich and complete our results. Third, our study was carried out in the context of a very particular epidemic crisis whose biological, epidemiological and socio-economical characteristics should be carefully considered before transposing our results and recommendations for another emerging infection. Fourth, the psychiatric services usually established in the three general hospitals where this study was performed probably influenced both organization and efficacy of these new support teams, thus leading to caution when it comes to generalize our conclusions to other territorial contexts. Fifth and last, the heterogeneity of the included mhPs regarding their profession, years of professional experience and usual department(s) of activity represents another limit to the transposition of our results to other medical settings. In particular, most of them – 14 out of 22 – were currently working with children and adolescents outside their period of activity within the MHSTs, which could imply differences of experience with mhPs usually caring for adults.

## Conclusion

The lived experience of mhPs redeployed on support teams for hospitalized COVID-19 patients and their families highlight both challenges and opportunities that health system disruption due to the pandemic represented for IC. This study points out that, in times of epidemic crisis, normative integration based on person- and family-centered approach is essential to guide integration of mental and physical health care in general hospital. In this perspective, our study promotes hospital and national policies both enabling and drawing from bottom-up mental health IC initiatives such as MSHTs implementation.
